# Multiplex PCR in Donor and Recipient Bronchoalveolar Lavage to Guide Early Antibiotic Prophylaxis Adaptation in Lung Transplantation: A Single-Center Cohort Study

**DOI:** 10.3390/jcm14238613

**Published:** 2025-12-04

**Authors:** Damien Barrau, Geoffrey Brioude, Alban Todesco, Erwan Mesdon, Benjamin Coiffard, Christophe Guervilly, Geoffray Agard, Florence Daviet, Benoit D’Journo, Jean-Marie Forel, Marc Leone, Pierre Mora, Sami Hraiech

**Affiliations:** 1 Service de Médecine Intensive—Réanimation, AP-HM, Hôpital Nord, 13015 Marseille, France; damien.barrau@ap-hm.fr (D.B.); erwan.mesdon@ap-hm.fr (E.M.); christophe.guervilly@ap-hm.fr (C.G.); geoffray.agard@ap-hm.fr (G.A.); florence.daviet@ap-hm.fr (F.D.); jean-marie.forel@ap-hm.fr (J.-M.F.); 2 Service de Chirurgie Thoracique, Transplantation Pulmonaire et Maladies de l’œsophage, AP-HM, Hôpital Nord, 13015 Marseille, France; geoffrey.brioude@ap-hm.fr (G.B.); alban.todesco@ap-hm.fr (A.T.); xavier.djourno@ap-hm.fr (B.D.); 3 Service de Pneumologie-Maladies Rares, AP-HM, Hôpital Nord, 13015 Marseille, France; benjamin.coiffard@ap-hm.fr; 4 Faculté de Médecine, Centre d’Etudes et de Recherche sur les Services de Santé et qualité de vie EA 3279, Aix Marseille Université, 13005 Marseille, France; 5 Service d’Anesthésie-Réanimation, Hôpital Nord, AP-HM, Aix Marseille Université, 13015 Marseille, Francepierre.mora@ap-hm.fr (P.M.)

**Keywords:** lung transplantation, antibiotic prophylaxis, multiplexPCR, bronchoalveolar lavage

## Abstract

**Background/Objectives**: International guidelines recommend the use of antibiotic prophylaxis for lung transplantation (LT). Although multiplex PCR (mPCR) has been shown to hasten antibiotic adaptation during pneumonia, its use to guide antibiotic prophylaxis in patients undergoing LT has not been described. We aimed to determine whether mPCR in bronchoalveolar lavage (BAL) in donor and recipient allows the early adaptation of antibiotic prophylaxis during LT. **Methods**: a retrospective, single-center study to evaluate the proportion of patients for whom mPCR (FilmArray Pneumonia Plus Panel^®^, Biomérieux (FAPP)) in the donor and recipient BAL resulted in an early modification of antibiotic prophylaxis. We also compared the time to results using mPCR and standard microbiology and the time spent with inadequate antibiotic prophylaxis. **Results**: Forty-one patients were included. Donor and recipient mPCR resulted in the early adaptation of antibiotic prophylaxis in 10 (24%) patients. Standard microbiology confirmed the results of mPCR in 90% of them. FAPP resulted in an antibiotic escalation based on donor (9/10) or recipient (1/10) BAL identification, mainly Group 3 Enterobacterales and non-fermenting Gram-negative bacilli. The time to results was 1.7 (1.5–2.4) h for mPCR vs. 74.3 (41.5–92.7) h for standard microbiology (*p* < 0.001) on donor BAL and 1.7 (1.5–2.4) h vs. 92.8 (48.4–112.9) h (*p* < 0.001) on recipient BAL. Patients with mPCR-based adaptation had a 71.9 (30.7–92.1) h reduction in the duration of inadequate antibiotic prophylaxis. **Conclusions**: mPCR in donor and recipient BAL during LT might lead to faster adaptation and a reduction in the time spent with inadequate antibiotic prophylaxis.

## 1. Introduction

Post-operative pneumonia significantly alters the prognosis of patients undergoing lung transplantation (LT) [1]. To prevent surgical site infections and early pneumonia, international guidelines recommend antibiotic prophylaxis for LT, but there is a high variability concerning the spectrum of antibiotics proposed and the duration of treatment [2,3,4]. Consequently, practices vary worldwide [5]. Antibiotic prophylaxis is based on the recipient’s colonization, which is often assessed several weeks before transplantation, raising the question of its adequacy at the time of surgery. It is also adapted to the donor’s infection or colonization, but complete microbiological identification is often not available for several days. This delay creates a risk of inadequate coverage during the period of maximal immunosuppression, exposing patients to severe infections.

FilmArray Pneumonia Plus Panel ^®^ (FAPP, Biomérieux, Marcy-l’Étoile, France) is a multiplex PCR (mPCR) technique that can quantitatively or semi-quantitatively detect an extensive panel of respiratory bacteria, viruses, and frequently encountered resistance genes [6]. Studies report a positive predictive value of 96.2% and a negative predictive value of 98.1% as well as excellent sensitivity and specificity compared with standard cultures [6]. The average time from sampling to delivery to the physician is around 4 h [7]. While its role in diagnosing pneumonia remains to be defined, mPCR has been associated with a faster adjustment of antibiotics in two recent large-scale randomized controlled trials (RCT). Indeed, its use in the diagnosis of pneumonia has been shown to significantly reduce the duration of inadequate antibiotic therapy (up to 39 h) and shorten the time to therapeutic change but without impact on de-escalation [8,9]. Data focusing specifically on LT patients remain scarce. When lower respiratory tract infection is suspected, FAPP on bronchoalveolar lavage (BAL) has been reported to provide faster results than conventional tests with a good concordance [10]. Although it is designed as a diagnosis tool, the accuracy and fast results of mPCR in respiratory samples make it a potentially useful tool for adjusting antibiotic prophylaxis during LT. Few data describe the use of FAPP in this indication. An observational study including 50 patients showed that FAPP performed on donor lung samples reduced the time taken to detect pathogens, although a significant number of bacteria were not detected by the panel [11]. Nevertheless, these data were limited to a microbiological description and test performance analysis. We report, here, a one-year experience of routinely using FAPP on both donor and recipient BALs to guide the early adaptation of antibiotic prophylaxis during LT. Our primary objective was to evaluate the proportion of patients for whom FAPP resulted in an adjustment to antibiotic prophylaxis and to describe the main pathogens involved in such cases. Secondary objectives were to compare time to result and the correlation between FAPP and standard microbiology. We hypothesized that the use of FAPP might reduce the duration of inadequate antibiotic prophylaxis.

## 2. Materials and Methods

### 2.1. Study Design and Population

We conducted a retrospective cohort study in Marseille (France) University Hospital lung transplantation center. Patients receiving a single or bilateral LT from 1 January to 31 December 2024 were included if a FAPP on the donor and/or the recipient BAL had been performed and the results were available on the electronic medical file. Patients for whom FAPP was not performed either in donor or in recipient respiratory samples, or for whom FAPP results were not available in the medical electronic file, were excluded. Patients expressing a refuse to the retrospective use of their medical data after information were not included according to French law.

### 2.2. Bacteriological Samples

Center protocol for LT includes a bacterial culture on BAL retrieved from the donor at the time of lung procurement (on donor site and brought back to the transplant center for analysis) and in the recipient 24 h after intensive care unit (ICU) admission. Direct examination and standard bacterial culture on BAL, blood culture, organ preservation liquid, explanted lung biopsy, and donor bronchial recut (segments of the donor bronchus resected at the time of procurement) are also routinely performed. From January 2024, the center protocol included, as part of routine care, a FAPP performed on BAL from donor and recipient.

For donors presenting pneumonia at the time of organ procurement, bacterial samples are made under antibiotics. For recipients, antibiotic prophylaxis is initiated in the operating room, meaning that all samples are collected under antibiotic exposure.

### 2.3. Antibiotic Prophylaxis Protocol

All antibiotic prophylaxis regimen are decided during multidisciplinary meetings (thoracic surgeons, pneumologists, intensivists, anesthesiologists, infectious diseases experts) before inscription on LT list. They are based on recipient bacterial colonization and underlying lung disease. Antibiotics are initiated preemptively and continued until microbiological results are available. Briefly, a third-generation cephalosporin is prescribed in patients without colonization and carried on upon culture sample results, for up to 7 days (or more for cystic fibrosis patients). In case of recipient bacterial colonization, antibiotic prophylaxis spectrum encompasses the identified bacteria.

Since the implementation of our local protocol using donor and recipient FAPP on BAL, anesthesiologists and ICU physicians are informed about FAPP results by the microbiology laboratory and adapt antibiotic prophylaxis if bacteria or resistance genes are present in at least one of the samples and not covered by the current antibiotic regimen. FAPP is used to guide early prophylactic adaptation because it provides rapid information on bacterial presence and resistance genes, allowing timely adjustment of therapy to reduce the duration of inadequate coverage. Adaptation is driven by local guidelines. No antibiotic discontinuation or de-escalation is allowed based solely on FAPP results, because the panel does not detect all potentially relevant pathogens and relying exclusively on it could expose patients to a risk of insufficient antimicrobial coverage in the early post-transplant period. Secondary adaptation or antibiotics withdrawal are driven by bacterial cultures.

### 2.4. Definitions

In our study, we defined an inadequate antibiotic prophylaxis as an antibiotic regimen that did not cover all the bacteria recovered during LT either in the donor or the recipient samples.

The duration of inadequate antibiotic prophylaxis corresponded to the time between the initiation of antibacterial prophylaxis and the modification of this treatment because of the identification of uncovered pathogens.

### 2.5. Data Collection

The following data were collected from the patients’ electronic medical file:

- Patients’ demographic characteristics and severity score at ICU admission [12,13]
- Microbiological documentation on bacteriological samples during operating room and during the 7 days after LT
- Time from bacteriological sample to results availability for physicians
- Antibiotic prophylaxis regimen (drugs, timing of modification if any, duration)
- Infections during the ICU stay including pneumonia (and documentation if any)
- Antibiotics treatment during the ICU stay (other than prophylaxis)
- Duration of mechanical ventilation, ICU and hospital length of stay, mortality at day 28 and day 90

### 2.6. Outcomes

The primary endpoint was the proportion of patients for whom the donor/recipient FAPP resulted in a modification of antibiotic prophylaxis before the results of standard microbiology were available (early adaptation).

Secondary endpoints included the total rate of patients for whom antibiotic prophylaxis was modified, time to bacteriological samples results, correlation between FAPP and cultures, sensitivity and specificity of FAPP (considering standard microbiology as the “gold standard”), time with inadequate antibiotic prophylaxis, patients for whom a hypothetical de-escalation would have been possible based on FAPP results, ventilator/healthcare-associated pneumonia (HAP/VAP) episodes, duration of invasive mechanical ventilation and ICU stay, and mortality within 28 and 90 days after LT.

### 2.7. Ethics Statement

Patients and their relatives were informed of the possible use of their medical data for retrospective studies and their opposition was researched. The study was approved by the French Intensive Care Society Ethics Committee (Commission d’Ethique de la SRLF, CE-SRLF 25-043) which waived the need for written consent according to the French legislation. The study was also declared and approved by our institution “Portail d’Accès aux Données de Santé, Assistance Publique-Hôpitaux de Marseille” (Registration number PADS24-208_dgr).

### 2.8. Statistical Analysis

Categorical variables were expressed as numbers and percentages. Continuous variables were presented as median and 25–75% interquartile range (IQR) and compared either using Wilcoxon’s rank sum test for two independent group comparisons or with the non-parametric Kruskal–Wallis test for multiple comparisons with Dunn post hoc test. Sensitivity and specificity of FAPP were calculated in donor and recipient samples. Sensitivity of FAPP was calculated by dividing the number of true positives (standard microbiology confirming FAPP results) by the sum of true positives and false negatives (bacteria detected in standard microbiology and not in the FAPP). Specificity was calculated by dividing the number of true negatives (no pathogen found in FAPP and sterile standard microbiology) by the sum of true negatives and false positives (pathogen detected by FAPP not confirmed by standard microbiology). The significance level was 0.05. All statistical tests and figures were performed with SPSS 20.0 software (IBM Corp, Armonk, NY, USA).

## 3. Results

**Figure 1:**
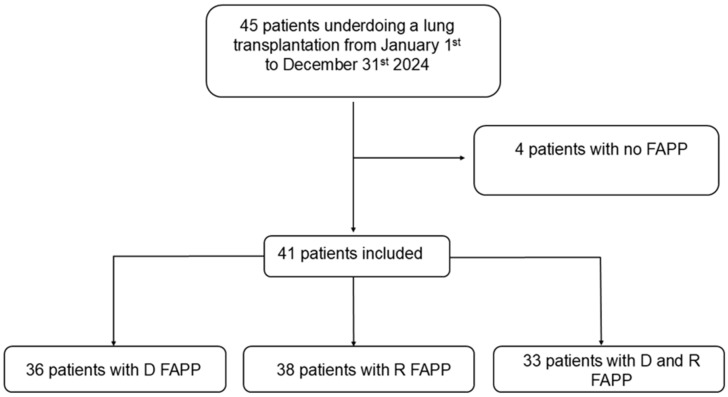
Study flowchart. D: donor. FAPP: FilmArray Pneumonia Plus Panel ^®^. R: recipient.

During the study period, 45 patients underwent an LT. Among them, 41 had a FAPP on the donor and/or recipient BAL and were therefore included in the study (Figure 1). Four patients had no FAPP either in donor or in recipient BAL because of logistical issues and were therefore excluded.

Patients’ characteristics at ICU admission are presented in Table 1. 

They were mainly men (63%) with a median age of 59 (47–63) years old. The main indication for LT was chronic obstructive pulmonary disease (44%), pulmonary fibrosis (41%), and cystic fibrosis or bronchiectasis (7%).

### 3.1. Primary Outcome

Donor and recipient FAPP resulted in an early adaptation of antibiotic prophylaxis in 10 (24%) patients. For 9 of these 10 patients (90%), bacterial culture secondarily confirmed the results of FAPP.

FAPP resulted in an antibiotic escalation based on donor (9/10) or recipient (1/10) BAL identification. For eight patients, escalation was due to pathogens not covered by the initial prophylaxis (five Group 3 Enterobacterales with inducible cephalosporinase, one *Pseudomonas aeruginosa*, and one *Acinetobacter* spp.), while in the two other cases, escalation was prompted by resistance (1 extensive spectrum beta-lactamase (ESBL)-producing Enterobacterale and 1 methicillin-resistant *Staphylococcus aureus* (MRSA)). The details of bacterial identification and antibiotic prophylaxis modification are described in Table 2 and in Figure S1.

Five patients (12%) had an antibiotic prophylaxis modification based on standard microbiology, resulting in an overall antibiotic prophylaxis change in fifteen (37%) patients.

### 3.2. Time to Sample Results and Antibiotic Prophylaxis Adaptation

**Figure 2:**
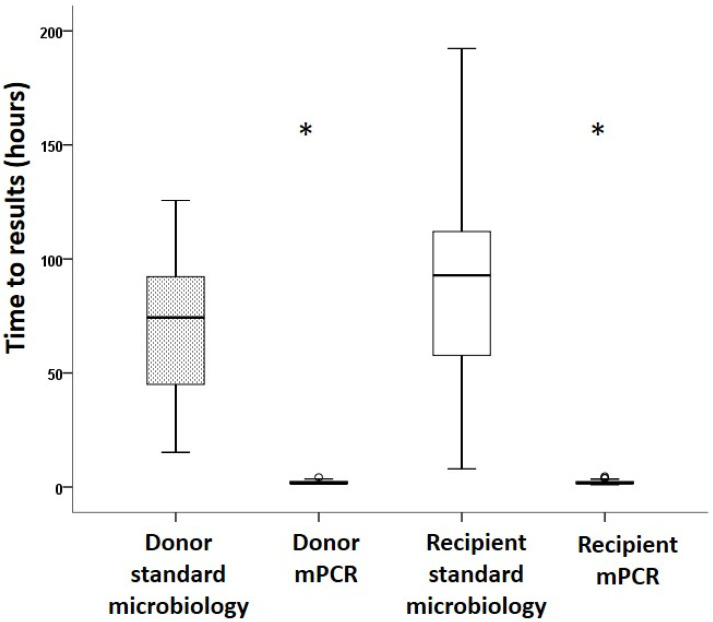
Comparison of FAPP and standard microbiology time to results on donor and recipient BAL. The box plot limits represent the 25th and 75th percentiles, and the bars represent the 5th and 95th percentiles. The median is represented as a horizontal line. Extremes values are represented by circles * *p* < 0.001 vs. standard microbiology.

Overall, the median time to sample results on donor BAL was 1.7 (1.5–2.4) hours (h) for FAPP vs. 74.3 (41.5–92.7) h for standard microbiology (*p* < 0.001). The median time to sample results on recipient BAL was 1.7 (1.5–2.4) h vs. 92.8 (48.4–112.9) h (*p* < 0.001) (Figure 2).

When focusing on patients with antibiotic prophylaxis adaptation (n = 15), the median time to antibiotic change was 2.4 (1.6–2.5) h when based on FAPP vs. 43.7 (20–85.9) h when based on standard microbiology (*p* = 0.01). Patients with FAPP-based adaptation had a 71.9 (30.7–92.1) h reduction in the duration of inadequate antibiotic prophylaxis compared to what would have been performed based on standard microbiology.

### 3.3. FAPP and Standard Microbiology Correlation

The sensitivity and specificity of FAPP were, respectively, 83% and 53% on donor samples and 91% and 44% on recipient samples.

FAPP and standard microbiology were exactly correlated in 8/32 (25%) patients for donor samples and in 5/31 (16%) for recipients, notably when all samples were sterile or in the case of monobacterial positive culture. FAPP identified more than one bacterium in 11/36 (31%) cases for donor BAL and in 8/38 (21%) cases for recipient BAL. For 20 (49%) patients, FAPP retrieved bacteria that were not found in culture. Standard microbiology identified bacteria not detected by the FAPP in seven patients (five Enterobacterales, five *S. anginosus* and one *M. luteus)*.

### 3.4. FAPP-Based Potential De-Escalation

In the 10 patients for which donor and recipient FAPP identified no bacteria, an early antibiotic interruption would have been possible for 6 (60%) of them for which standard microbiology was sterile.

### 3.5. Clinical Outcomes

Main outcomes are described in Table 3.

HAP/VAP occurred in 11 (27%) patients during the ICU stay and 9 of them (22%) had a third-generation cephalosporin-resistant Gram-negative bacilli ICU acquired infection. The median duration of MV after LT was 4 (1–11) days. The mortality rate at day 90 was 9.8%.

## 4. Discussion

We report here the first pragmatic series describing the use of routine donor and recipient FAPP on BAL as a tool for early adaptation of antibiotic prophylaxis during LT. We found that FAPP enabled treatment to be modified for 10/41 (24%) patients, with this adaptation occurring in less than 3 h. The documentation retrieved by FAPP was confirmed for 90% of patients, leading to a reduction in time with inadequate antibacterial prophylaxis of 3.2 days.

The role of mPCR in improving the treatment of pneumonia remains debated. However, when focusing on recently published RCT, mPCR has been shown to reduce the duration of inadequate therapy [8,9]. It has been shown to enable faster antibiotic escalation for Gram-negative or Gram-positive bacteria and faster antibiotic de-escalation for Gram-positive bacteria only. In a recent trial focusing on HAP and VAP, FAPP enhanced antibiotic stewardship but failed to demonstrate non inferiority for clinical cure as compared to standard of care [14].

Among LT patients, the use of mPCR has mainly been described for the diagnosis of pneumonia. In a prospective series of 60 LT patients [10] with low respiratory tract infection who underwent a BAL, mPCR allowed to significantly reduce the time to bacterial and viral identification (2.3 (2–2.8) h for mPCR vs. 23.4 h (21.1–62, *p* < 0.001) and 25.2 h (22.8–69.5, *p* < 0.001), for virology and microbiology, respectively) as well as the time to clinical decision and resulting treatment modification (2.8 (IQR 2.2–44) for mPCR versus 28.1 h (23.1–70.6) and 32.6 h (24.6–70.9) for overall conventional virology and microbiology, respectively (both *p* < 0.001)). Antibiotic therapies based on mPCR results were maintained in 90% of cases. In our cohort, 90% of bacteria identified in FAPP, and followed by an early adaptation antibiotic prophylaxis, were confirmed in conventional culture.

The evaluation of FAPP for the rapid detection of bacteria in donor BAL at different time points has been described in one series of 50 patients [11]. The authors reported a shorter time to detection of lung pathogens in donated lungs. However, a subsequent number of pathogens were not detected because they were not included in the panel, mainly fungi, coagulase-negative *Staphylococci* and *S. maltophilia.* However, the authors did not evaluate the impact of FAPP on antibiotic management. We found herein that, as compared with standard microbiology, FAPP had high sensitivity but moderate specificity in donor and recipient samples. These results are not consistent with those reported in studies focusing on the diagnosis of pneumonia in LT patients. Kayser et al. [10] reported a sensitivity and a specificity for included bacteria of 58% and 100%, respectively. Drick et al. [15] found a sensitivity of 66% and a specificity of 100%. In our cohort, the frequent identification of more than one bacterium in donor BAL FAPP might explain this discrepancy. Polymicrobial FAPP positivity has been described as a common cause of discordance with culture [6,7], especially when respiratory samples are retrieved under antibiotics. This situation frequently occurs in donors who often suffer from pneumonia. Antibiotics may suppress growth in culture, while still allowing FAPP to detect bacterial DNA, which can lead to apparent false positives. The clinical significance of bacteria identified by FAPP but not by culture in polymicrobial samples remains unclear, challenging the ‘gold standard’ position of culture in some situations. The relevance of a positive FAPP result with a low bacterial threshold (104 copies/mL) that is not retrieved in culture remains unclear. Potential false positives should be treated with caution as they pose a risk of antibiotic overexposure in patients, which could contribute to the emergence of antibiotic resistance.

The integration of FAPP to guide antibiotic prophylaxis during LT, currently under evaluation, has been proposed in a recently published algorithm (16) based on recipient and donor respiratory samples. Our experience of using FAPP clinically for one year showed that most antibiotic changes were based on donor documentation. It is possible that FAPP for recipients might be useful for patients colonized with multi-drug-resistant bacteria before LT. In our cohort, two thirds of patients had no prior bacterial colonization.

Early antibiotics discontinuation, based on donor’s and recipient’s negative FAPP might have been safely possible in only 60% of patients and should not be recommended. Considering the bacteria outside the panel (mainly Group 3 Enterobacterales such as *M. morganii*, *H. alvei*, *Citrobacter* spp. or non-fermenting Gram-negative bacteria such as *S. maltophilia*), it seems reasonable to use FAPP as a tool for the early detection of these bacteria. The possibility of narrowing the spectrum of antibiotic prophylaxis based on FAPP still deserves investigation. Given the panel’s inability to detect fungal pathogens, FAPP must be incorporated into the overall strategy for guiding antimicrobial prophylaxis after LT.

Our study has some limitations. Firstly, the retrospective design may have introduced biases. However, we adhered closely to the protocol, excluding only four patients because at least one FAPP was not collected in donor or recipient. Secondly, we chose to perform BAL on the recipient after LT rather than on the explanted lungs in the operating room. Similar procedures have been reported [16] and rapid colonization of the newly implanted lungs from the recipient’s upper airway is common in cases of colonization. Lastly, it is not possible to determine whether early modification of antibiotics based on FAPP resulted in a change in clinical outcomes. These preliminary data will have to be confirmed in controlled trials, especially to evaluate the impact of our strategy on the rate of HAP/VAP, the emergence of third generation cephalosporin-resistant Gram-negative bacilli colonization, and infection and carbapenem use.

## 5. Conclusions

We present herein the first study investigating the routine use of FAPP in donor and recipient samples to guide antibiotic prophylaxis during LT. In our experience, FAPP resulted in an antibiotic modification in almost one quarter of patients, exclusively represented by an earlier consideration of uncovered bacteria. This resulted in a significant reduction in the duration of inadequate antibiotic prophylaxis compared with standard microbiology. While these findings are promising, they need to be confirmed in prospective multicenter studies to better define the exact role of FAPP in this setting and include a cost-effectiveness evaluation.

## Figures and Tables

**Table 1:** Patients’ characteristics at ICU admission.

Age, years, median (IQR)	59 (47–63)
Male gender, n (%)	26 (63)
Ischemic heart disease, n (%)	10 (24)
Pulmonary hypertension, n (%)	31 (76)
Chronic renal failure, n (%)	11 (27)
Diabetes mellitus, n (%)	7 (17)
History of tobacco use, n (%)	22 (54)
Immunocompromised status before LT, n (%)	20 (49)
Indication for LT, n (%)	
COPD	18 (44)
Pulmonary fibrosis	17 (41)
CF or bronchiectasis	3 (7)
CLAD	3 (7)
Recipient bacterial lung colonization before LT, n (%)	14 (34)
SAPS 2, median IQR	39 (29–46)
SOFA, median IQR	7 (6–8)
Grad 3 primary allograft dysfunction, n (%)	9 (22)

CF: cystic fibrosis. CLAD: chronic lung allograft dysfunction. COPD: chronic obstructive pulmonary disease. ICU: intensive care unit. LT: lung transplantation. SAPS2: Simplified Acute Physiology Score 2. SOFA: sequential organ failure score.

**Table 2:** Antibiotic prophylaxis modifications during or after LT.

No modification, n (%)	26 (63)
De-escalation, n (%)	0
Escalation, n (%)	15 (37)
FAPP-based modification n (%)	10 (24)
*** Identification justifying the modification***	
Gram-positive bacteria:	1
MRSA	1
MRCNS	0
Gram-negative bacilli:	9
ESBL-EB	1
Group 3 EB with inducible cephalosporinase	5
Non-fermentant GNB:	
* P. aeruginosa*	1
* Acinetobacter* spp.	1
Intra-cellular: *M. pneumoniae*	1
Standard microbiology (Direct Examination/Culture)-based modification, n (%)	5 (12)
*** Sample site justifying the modification***	
* BAL or tracheal aspirate*	2
* Lung biopsy*	1
* Lung conservation liquid*	2
*** Identification justifying the modification***	
Gram-positive bacteria:	2
MRSA	0
MRCNS	2
Gram-negative bacilli:	3
ESBL-EB	0
Group 3 EB with inducible cephalosporinase	1
Non-fermentant GNB	2
* P. aeruginosa*	0
* Acinetobacter* spp.	1
* S. maltophilia*	1
Initial antibiotic prophylaxis, n *:	
Cefotaxime	11
Piperacillin–tazobactam	4
Post-adaptation antibiotic prophylaxis, n *	
Piperacillin–tazobactam	3
Cefepime	4
Carbapenem	3
Fluoroquinolon in combination	1
Linezolid in combination	3
Cotrimoxazole in combination	1
Time to antibiotic prophylaxis adaptation, hours, median (IQR) *	
FAPP-based	2.4 (1.6–2.5)
Direct examination/Culture-based	43.7 (20–85.9)

* For patients with antibiotic prophylaxis modification (n = 15). BAL: bronchoalveolar lavage. EB: Enterobacterales. ESBL: extended spectrum beta-lactamase. FAPP: FilmArray Pneumonia Plus Panel ^®^. GNB: Gram-negative bacilli. IQR: interquartile range. MRCNS: methicillin-resistant coagulase negative *Staphylococcus*. MRSA: methicillin-resistant *Staphylococcus aureus.*

**Table 3:** Patients’ outcomes during the ICU stay.

Donor pneumonia, n (%)	18 (44)
HAP/VAP, n (%)	11 (27)
C3G-R GNB acquired colonization, n (%)	6 (15)
C3G-R GNB acquired infection, n (%)	9 (22)
Carbapenem use, n (%)	8 (20)
Duration of mechanical ventilation, days, median (IQR)	4 (1–11)
Ventilator free days at day 28, days, median (IQR)	24 (17–27)
Duration of ICU stay, days, median (IQR)	11 (9–22)
Mortality at day 28, n (%)	1 (2.4)
Mortality at day 90, n (%)	4 (9.8)

C3G-R: Third generation cephalosporin-resistant. GNB: Gram-negative bacilli. HAP: healthcare associated pneumonia. ICU: intensive care unit. IQR: interquartile range. VAP: ventilator associated pneumonia.

## Data Availability

The data presented in this study are available on request from the corresponding author due to legal and ethical reasons.

## Supplementary Materials

The following supporting information can be downloaded at: https://www.mdpi.com/article/10.3390/jcm14238613/s1, Figure S1: Donor and recipient BAL bacterial documentation retrieved in standard microbiology, FAPP, and both techniques. FAPP: FilmArray Pneumonia Plus Panel ^®^.

## References

[1] Walti L.N., Ng C.F., Mohiuddin Q., Bitterman R., Alsaeed M., Klement W., Martinu T., Sidhu A., Mazzulli T., Donahoe L. (2024). Hospital-Acquired and Ventilator-Associated Pneumonia Early After Lung Transplantation: A Prospective Study on Incidence, Pathogen Origin, and Outcome. Clin. Infect. Dis..

[2] Abbo L.M., Grossi P.A., AST ID Community of Practice (2019). Surgical site infections: Guidelines from the American Society of Transplantation Infectious Diseases Community of Practice. Clin. Transplant..

[3] Marczin N., de Waal E.E., Hopkins P.M., Mulligan M.S., Simon A., Shaw A.D., Van Raemdonck D., Neyrinck A., Gries C.J., Algotsson L. (2021). International consensus recommendations for anesthetic and intensive care management of lung transplantation. An EACTAIC, SCA, ISHLT, ESOT, ESTS, and AST approved document. J. Heart Lung Transplant..

[4] Righi E., Mutters N.T., Guirao X., del Toro M.D., Eckmann C., Friedrich A.W., Giannella M., Kluytmans J., Presterl E., Christaki E. (2023). ESCMID/EUCIC clinical practice guidelines on perioperative antibiotic prophylaxis in patients colonized by multidrug-resistant Gram-negative bacteria before surgery. Clin. Microbiol. Infect..

[5] Coiffard B., Prud’hOmme E., Hraiech S., Cassir N., Le Pavec J., Kessler R., Meloni F., Leone M., Thomas P.A., Reynaud-Gaubert M. (2020). Worldwide clinical practices in perioperative antibiotic therapy for lung transplantation. BMC Pulm. Med..

[6] Buchan B.W., Windham S., Balada-Llasat J.M., Leber A., Harrington A., Relich R., Murphy C., Dien Bard J., Naccache S., Ronen S. (2020). Practical Comparison of the BioFire FilmArray Pneumonia Panel to Routine Diagnostic Methods and Potential Impact on Antimicrobial Stewardship in Adult Hospitalized Patients with Lower Respiratory Tract Infections. J. Clin. Microbiol..

[7] Crémet L., Gaborit B., Bouras M., Drumel T., Guillotin F., Poulain C., Persyn E., Lakhal K., Rozec B., Vibet M.-A. (2020). Evaluation of the FilmArray® Pneumonia Plus Panel for Rapid Diagnosis of Hospital-Acquired Pneumonia in Intensive Care Unit Patients. Front. Microbiol..

[8] Darie A.M., Khanna N., Jahn K., Osthoff M., Bassetti S., Osthoff M., Schumann D.M., Albrich W.C., Hirsch H., Brutsche M. (2022). Fast multiplex bacterial PCR of bronchoalveolar lavage for antibiotic stewardship in hospitalised patients with pneumonia at risk of Gram-negative bacterial infection (Flagship II): A multicentre, randomised controlled trial. Lancet Respir. Med..

[9] Virk A., Strasburg A.P., Kies K.D., Donadio A.D., Mandrekar J., Harmsen W.S., Stevens R.W., Estes L.L., Tande A.J., Challener D.W. (2024). Rapid multiplex PCR panel for pneumonia in hospitalised patients with suspected pneumonia in the USA: A single-centre, open-label, pragmatic, randomised controlled trial. Lancet Microbe.

[10] Kayser M.Z., Seeliger B., Valtin C., Fuge J., Ziesing S., Welte T., Pletz M.W., Chhatwal P., Gottlieb J. (2022). Clinical decision making is improved by BioFire Pneumonia Plus in suspected lower respiratory tract infection after lung transplantation: Results of the prospective DBATE-IT study. Transpl. Infect. Dis..

[11] Nguyen A., Chen J., Isaza E., Panchal N., Deiter F., Hoover J., Trinh B., Hays S.R., Golden J.A., Singer J.P. (2023). Biofire pneumonia panel in lung donors: Faster detection but limited pathogens. Transpl. Infect. Dis..

[12] Le Gall J.-R., Lemeshow S., Saulnier F. (1993). A new Simplified Acute Physiology Score (SAPS II) based on a European/North American multicenter study. JAMA.

[13] Vincent J.L., Moreno R., Takala J., Willatts S., De Mendonça A., Bruining H., Reinhart C.K., Suter P.M., Thijs L.G. (1996). The SOFA (Sepsis-related Organ Failure Assessment) score to describe organ dysfunction/failure. On behalf of the Working Group on Sepsis-Related Problems of the European Society of Intensive Care Medicine. Intensive Care Med..

[14] Enne V.I., Stirling S., Barber J.A., High J., Russell C., Brealey D., Dhesi Z., Colles A., Singh S., Parker R. (2025). INHALE WP3, a multicentre, open-label, pragmatic randomised controlled trial assessing the impact of rapid, ICU-based, syndromic PCR, versus standard-of-care on antibiotic stewardship and clinical outcomes in hospital-acquired and ventilator-associated pneumonia. Intensive Care Med..

[15] Drick N., Seeliger B., Greer M., Bollmann B.A., Ziesing S., Welte T., Gottlieb J. (2018). DNA-based testing in lung transplant recipients with suspected non-viral lower respiratory tract infection: A prospective observational study. Transpl. Infect. Dis..

[16] Lombardi A., Mangioni D., Renisi G., Fumagalli J., Morlacchi L., Rosso L., Palleschi A., Rossetti V., Panigada M., Abbruzzese C. (2025). Dealing with Antibiotic Prophylaxis in Lung Transplantation in the Era of Multidrug Resistance: The Milano Algorithm. Transplant. Proc..

