# Supplementary figures and images for: Multiplex PCR in Donor and Recipient Bronchoalveolar Lavage to Guide Early Antibiotic Prophylaxis Adaptation in Lung Transplantation: A Single-Center Cohort Study
Source: J Clin Med. 2025 Dec 4;14(23):8613. doi: 10.3390/jcm14238613 (PMC12693403; doi:10.3390/jcm14238613)

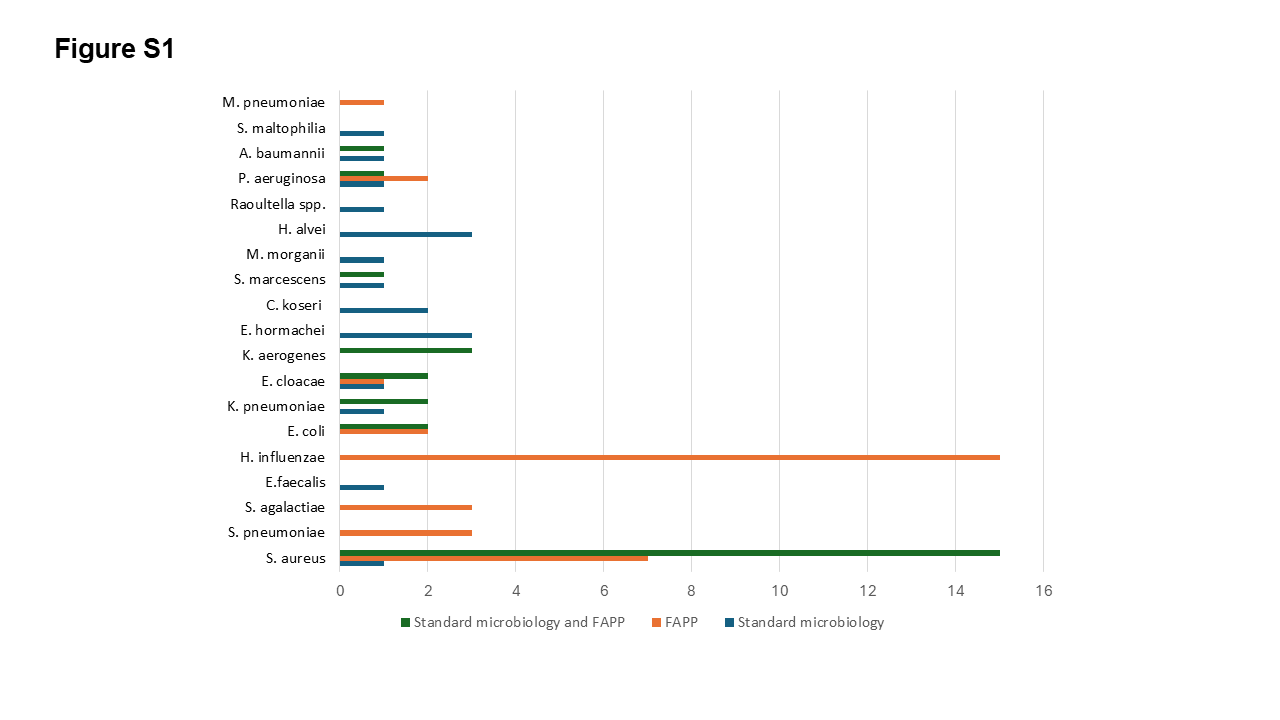

Supplement: Supplementary file 1 [file jcm-14-08613-s001.zip › jcm-3997115-supplementary.TIF]
